# Interobserver variability in lymph node evaluation with endoscopic ultrasonography in cholangiocarcinoma

**DOI:** 10.1055/a-2577-5449

**Published:** 2025-05-12

**Authors:** David Michaël de Jong, Daniëlle Roosterman, Marco J. Bruno, Lydi M.J.W. van Driel, Wim J. Lammers

**Affiliations:** 1 Gastroenterology and Hepatology, Erasmus MC University Medical Center Rotterdam, Rotterdam, Netherlands

**Keywords:** Endoscopic ultrasonography, Biliary tract, Fine-needle aspiration/biopsy, Tissue diagnosis

## Abstract

**Background and study aims:**

Accurate preoperative lymph node (LN) assessment is crucial for patients with intrahepatic cholangiocarcinoma (iCCA) and perihilar cholangiocarcinoma (pCCA) because presence of LN metastases significantly reduces survival rates and can contraindicate surgical resection. Endoscopic ultrasound (EUS) provides a reliable method for LN assessment with the advantage of enabling tissue acquisition for pathological confirmation. This study aimed to assess interobserver agreement among endosonographers in evaluating LN characteristics in patients with iCCA and pCCA.

**Methods:**

A cross-sectional survey study was conducted among 24 endosonographers. Participants reviewed 42 EUS images from iCCA and pCCA patients, classifying LNs based on six characteristics (demarcation, shape, echogenicity, homogeneity, suspiciousness, and need to retrieve tissue). Interobserver agreement was determined using Light’s kappa statistics. Accuracy, sensitivity, and specificity in identifying malignant LNs were calculated.

**Results:**

Overall kappa values indicated moderate to fair agreement on LN characteristics, with Kappa values of 0.24 for demarcation, 0.45 for shape, 0.38 for echogenicity, 0.52 for homogeneity, and 0.36 for suspiciousness. Overall accuracy of endosonographers in correctly identifying malignant LNs was 62%, with individual accuracy ranging from 44 to 75%. Sensitivity was 60% (range: 29%–90%) and specificity was 64% (range: 28%–89%).

**Conclusions:**

Endosonographic assessment of LN morphology and characterization demonstrates considerable variability among endosonographers. Thus, there is a clear need for standardization in preoperative LN evaluation, including establishing consensus about when to perform tissue acquisition, based on objective criteria such as short-axis diameter. Further research is required to refine and optimize these guidelines.

## Introduction

Cholangiocarcinoma (CCA) is a malignancy of the biliary epithelium, classified by tumor location into intrahepatic (iCCA), perihilar (pCCA), and distal (dCCA) subtypes 1 . Notably, CCA is often diagnosed at an advanced stage, limiting curative treatment options 2 3 4 . Only 15% of patients are eligible for curative resection 5 6 7 . Likelihood of resection is influenced by tumor characteristics such as vascular involvement, local extension, and presence of metastases 8 .

Lymph node metastases (LNM) have a profound impact on survival, particularly in iCCA and pCCA, reducing median survival from 60 months to 20 months for iCCA and from 31 to 42 months to 13 months for pCCA 9 10 11 12 . Therefore, accurate preoperative evaluation of LN involvement is essential. Cross-sectional imaging modalities like computed tomography and magnetic resonance imaging have limited sensitivity for detecting LNM 13 . Endoscopic ultrasound (EUS), however, offers the advantage of tissue acquisition (EUS-TA) through fine-needle aspiration or biopsy, potentially preventing unnecessary invasive laparotomies 14 15 16 17 18 .

Several predictors of malignancy among pancreato-biliary malignancies have been proposed, including round shape, homogeneity, size > 10 millimeter (mm), sharply demarcated borders, and hypoechogenicity 19 20 . However, interobserver variability among endosonographers in assessing these characteristics is poorly understood, particularly in CCA 21 22 . This study aimed to assess interobserver agreement in EUS-based LN evaluation in patients with iCCA and pCCA and to determine the accuracy of identifying malignant LNs based on morphology.

## Methods

### Study design

This study employed a cross-sectional survey design involving endosonographers from the QUEST consortium (QUality in EndoSonography Team, established in 2015 23 ) as well as endosonographers participating in the ongoing Preoperative Evaluation of Lymph nodes in Cholangiocarcinoma (POELH) trial (Clinicaltrials.gov NCT05678218). Study reporting was checked according to the Checklist for Reporting Of Survey Studies (CROSS) (Supplementary File) 24 . The study protocol was approved by the Medical Ethics Committee at the Erasmus MC University Medical Center (MEC-2023–0281, date of approval: 05–06–2023). A total of 145 endosonographers from both peripheral and academic centers were invited to participate, all with experience in using EUS to evaluate LNs. Because CCA treatment is centralized in the Netherlands in a total of seven academic hospitals, only academic endosonographers performed preoperative LN staging in the setting of CCA. All participants were sent a personal link (single-use link) with access to a website to review EUS still images of LNs and complete the survey through the web-based database Castor 25 . The survey was open for completion during a time period of approximately 3 months, without prior sample size calculation.

### EUS image set

EUS images were collected during the aforementioned POELH trial and informed consent was retrieved from these patients. All EUS examinations were performed using the EG38-J10-UT Pentax scope. In total, 42 images from both benign and malignant LNs from different patients were selected. All images were then de-identified, and were randomly ordered in the survey.

In the POELH trial, which enrolled patients in six academic hospitals in the Netherlands and one center in Belgium, patients with presumed resectable perihilar, intrahepatic, and mid-common bile duct (CBD) CCA underwent preoperative EUS focusing on LN staging. To do so, a strict protocol was followed to systematically check both regional and extraregional LNs. In addition, all LNs with a short-axis > 5 mm were punctured, whenever safely possible. Images were taken of all identified LNs during EUS assessment. Regional and extraregional LN were defined according to the 8 ^th^ edition of the American Joint Committee on Cancer staging manual 26 . In short, for pCCA, regional LNs consisted of LNs at the liver hilum, cystic duct, common bile duct, hepatic artery, and portal vein, and extraregional LNs consisted of LNs at the peri-aortic region, peri-caval region, superior mesenteric artery, and celiac trunk. For iCCA, regional LNs were defined differently than for iCCA located in the left or right hemi-liver. Extraregional LNs for both iCCA sides were LN at the peri-aortic region, peri-caval region, and celiac trunk.

### Survey

The survey consisted of two sections (Supplementary File). In the first part, background information about the endosonographer was collected by requesting the following information: healthcare institution of employment (general or academic), total number of EUSs performed independently per year, and experience with EUS in the context of CCA. Level of expertise was classified as a composite endpoint of > 1000 procedures with > 200 to 300 EUSs per year with > 5 years of experience, mostly in line with the European Society of Gastrointestinal Endoscopy guidelines on EUS training curriculum 27 .

**Fig. 1:**
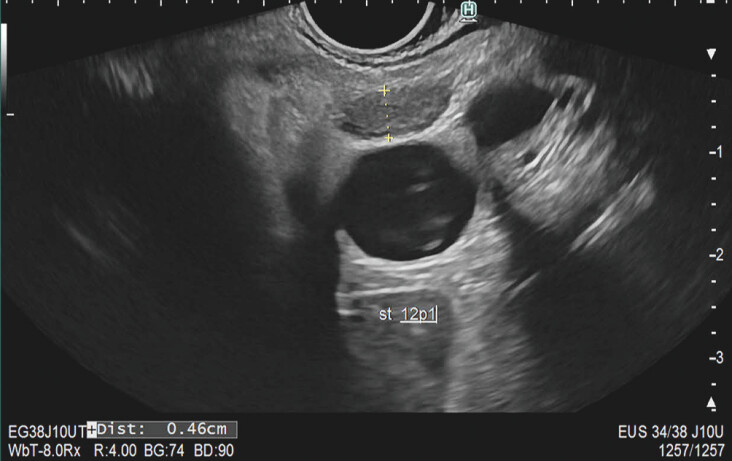
Lymph node station 12P1 at the portal vein. Surgery showed iCCA with benign LN.

**Fig. 2:**
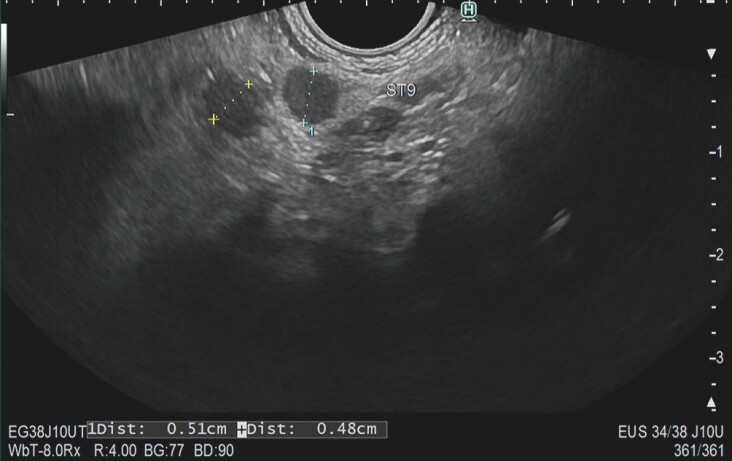
Lymph node station 9 at the celiac trunk. Presumed pCCA with EUS-TA (blue LN) showing LNM precluding surgery.

In the second part, 42 EUS images were shown. Examples are shown in Fig. 1 and Fig. 2 . Information was provided about the type of CCA, short-axis of the LN in mm, and location of the LN station, according to the 3rd edition of the Japanese Society of Hepato-Biliary-Pancreatic Surgery classification of biliary tract cancers 28 . Participants had to give their opinion about six LN characteristics for each particular LN (Supplementary File). The following characteristics were requested in multiple choice: demarcation of the LN, echogenicity, homogeneity, shape, whether they suspected the LN to be malignant, and if so, whether they would perform tissue acquisition or not.

### Objectives

The primary objective of this study was to assess the level of agreement among endosonographers regarding EUS-based evaluation of LN characteristics in patients with presumed resectable iCCA or pCCA. The secondary objective was to evaluate the accuracy of endosonographers in differentiating between benign and malignant LNs based on EUS characteristics. Analysis was stratified by participant experience and institutional setting.

### Statistical analysis

Interobserver agreement was quantified using Light’s kappa statistics with 95% confidence intervals (CI) calculated for each LN characteristic 29 . Subgroup analyses were performed to compare agreement between experienced and less experienced endosonographers, as well as between academic and general hospital practitioners. Inter-rater reliability (IRR) was performed to evaluate consistency in assigning LN characteristics 30 . Kappa values for each coder pair were averaged to provide an overall IRR index, with bootstrapping employed to calculate 95% CIs using 1000 replicates.

To calculate a kappa value, it is essential that each rater demonstrated variability in their responses. When a rater provided repetitive answers across all questions (i.e. all LNs were rated as having sharp demarcation), the rater was excluded from the analysis, in accordance with the assumptions of kappa analysis. Kappa values were computed for each pair of raters, and these values were subsequently averaged to provide an overall index of IRR 29 . To quantify uncertainty of the estimated kappa statistics, a bootstrapping method was employed generating a 95% CI using 1000 bootstrap replicates. Kappa statistics and bootstrap replicates were calculated using the “vcd” and “boot” package in R, version 4.2.2. Non-response error was not addressed, as only completely filled in surveys were used.

Kappa statistics were interpreted according to Landis and Koch’s criteria: value < 0.4 indicated fair agreement, 0.4 to 0.6 moderate agreement, 0.6 to 0.8 substantial (good) agreement, and values > 0.8 almost perfect agreement 31 .

Overall accuracy of identifying malignant LNs was calculated by constructing 2×2 tables comparing suspicious/non-suspicious findings with pathologically confirmed malignant/benign outcomes. Responses from endosonographers who answered "I don’t know" were excluded. All responses with “Other” were assigned to suspicious/non-suspicious accordingly. Sensitivity, specificity, and individual endosonographer accuracy were also calculated. LNs without pathological confirmation were excluded from the accuracy analysis.

## Results

### Characteristics of endosonographers and EUS images

Of the 145 endosonographers invited to participate, 24 (16.6%) completed the survey. Details regarding their experience and expertise are summarized in Table 1 . Half of the endosonographers conducted their procedures in academic hospitals, and 13 (54.2%) reported that they had independently performed more than 1000 EUS.

**Table:** **Table 1** Baseline characteristics of participating endosonographers **.**

Characteristics	n (%)
Endoscopist sex	
Male	15 (62.5)
Female	9 (37.5)
Type of hospital	
Academic	12 (50.0)
General (teaching and non-teaching)	12 (50.0)
Experience with EUS in setting of CCA	
Yes	12 (50.0)
No	12 (50.0)
Number of EUSs performed per year	
0–50	3 (12.5)
50–100	6 (25.0)
100–200	8 (33.3)
200–300	4 (16.7)
>300	3 (12.5)
Lifetime number of EUSs performed independently	
<400	2 (8.3)
400–1000	9 (37.5)
>1000	13 (54.2)
CCA, cholangiocarcinoma; EUS, endoscopic ultrasound; LN, lymph node.	

Characteristics of the 42 collected LNs are described in Table 2 . The majority of patients had iCCA or pCCA without underlying liver disease. Only 9.5% of patients had CCA in the context of primary sclerosing cholangitis (PSC). Of all reported LNs, 54.8% was defined as regional and 45.2% as extraregional. Median short-axis diameter of all LNs was 6.0 mm (interquartile range [IQR] 4.6–8.0). Only two LNs were >10 mm. Pathology results were available for 29 of 42 LNs (69.1%).

**Table:** **Table 2** Characteristics of included LNs.

Characteristics	Total (n = 42)	pCCA (n = 24)*	iCCA (n = 17)
Regional (vs. extraregional) – n (%)	23 (54.8)	17 (70.8)	6 (35.3)
PSC diagnosis – n (%)	4 (9.5)	2 (8.3)	2 (11.8)
Short-axis diameter <5 mm – n (%)	13 (31.0)	8 (33.3)	5 (29.4)
Short-axis diameter >10 mm – n (%)	2 (4.8)	0 (0)	2 (11.8)
Short-axis diameter – median (IQR), mm	6.0 (4.6–8.0)	5.6 (4.3–8.0)	5.9 (4.7–8.2)
EUS-TA performed – n (%) LNM	20 (47.6) 10 (23.8)	7 (29.2) 1 (4.2)	13 (76.5) 9 (21.4)
Surgical confirmation – n (%) LNM	10 (23.8) 1 (2.4)	9 (37.5) 1 (4.2)	1 (5.9) 0 (0)
Combined confirmation – n (%) LNM	29 (69.0) ^†^ 11 (26.2)	15 (62.5) ^†^ 2 (8.3)	14 (82.4) 9 (52.9)
EUS-TA, endoscopic ultrasound-guided tissue acquisition; iCCA, intrahepatic cholangiocarcinoma; IQR, interquartile range; LNM, lymph node metastasis; pCCA, perihilar cholangiocarcinoma; PSC, primary sclerosing cholangitis. *One patient with two LNs was included in the survey. ^†^ One LN had benign EUS-TA, but surgery showed malignancy.			

### Interobserver agreement

Overall diagnostic Light’s kappas were as follows: 0.24 (95% CI 0.17–0.34) for demarcation, 0.45 (95% CI 0.38–0.54) for shape, 0.38 (95% CI 0.32–0.46) for echogenicity, 0.52 (95% CI 0.42–0.64) for homogeneity, 0.36 (95% CI 0.27–0.45) for suspicion for malignancy, and 0.27 (95% CI 0.21–0.33) for the decision to perform an EUS-TA. For the kappa associated with “echogenicity”, two endosonographers were excluded due to lack of variability in their assessments. Similarly, for the kappa related to performing an EUS-TA, one endosonographer was excluded for the same reason. Sensitivity analysis for LN in which pathology was available revealed no significant differences in kappa values, as shown in the Supplementary File.

### Subgroup analyses

**Fig. 3:**
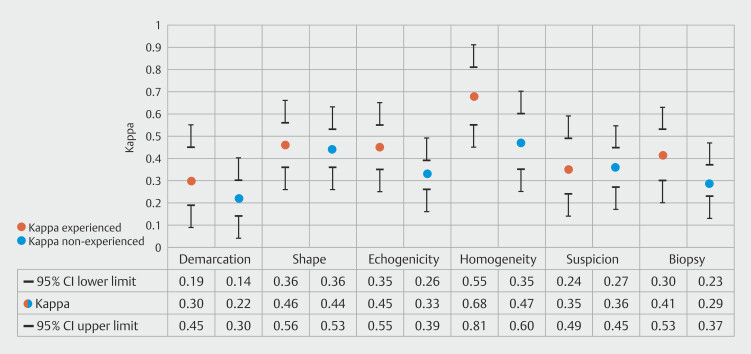
Kappa values per LN characteristic of experienced vs non-experienced endosonographers. CI, confidence interval.

**Fig. 4:**
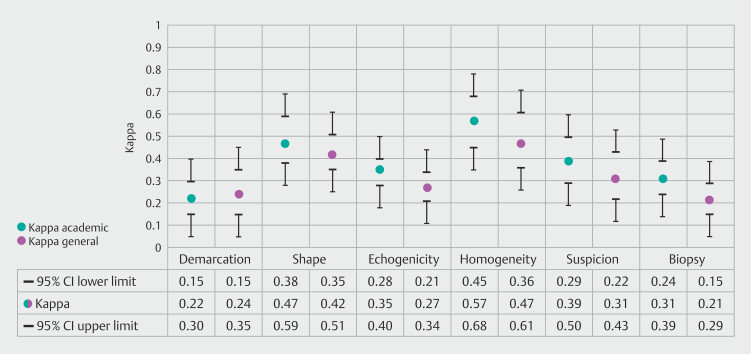
Kappa values per LN characteristic of endosonographers working in academic and general hospitals. CI, confidence interval.

Subgroup analyses comparing experienced vs. non-experienced endosonographers and endosonographers from academic vs. general hospital are presented in Fig. 3 and Fig. 4 , respectively. These analyses indicate a trend in which experienced endosonographers demonstrated higher kappa values across the six lymph node characteristics, a pattern that was similarly reflected in the comparison between endosonographers working in academic versus general hospital settings.

### Suspicion

Overall accuracy in correctly identifying a malignant LN was 62%, ranging from 44% to 75%. Overall sensitivity was 60%, whereas individual endosonographers exhibited a sensitivity range with a lower bound of 29% and an upper bound of 90%. Likewise, overall specificity was 64%, with a range of 28% to 89%.

## Discussion

Preoperative LN assessment using EUS is of utmost importance for patients with presumed resectable CCA because LNM significantly worsen prognosis, even when curative surgery is attempted. Accurate preoperative LN evaluation, therefore, can help prevent unnecessary surgical interventions that might not improve survival and could potentially diminish patient quality of life. This study showed limited interobserver agreement among endosonographers regarding assessment of LN morphology in CCA patients, as well as in accurate identification of malignant LNs.

Findings from this study align with previous research examining EUS performance in LN evaluation and description of important characteristics. For instance, Takasaki et al. reported fair to moderate interobserver agreement for shape, demarcation, and echogenicity, which is consistent with results from this study 21 . Similarly, other studies on LN assessment, although not specifically focused on CCA, have shown moderate and fair agreement on the same LN features 32 .

Subgroup analysis revealed slightly higher but not statistically significant agreement among experienced endosonographers compared with their less experienced counterparts. This finding is consistent with previous studies, which also reported that even endosonographers with > 5000 EUS procedures were not unanimous in their assessment 22 . This underscores the need for standardized and unified approaches to LN assessment, because current variations in interpretation may result in different LNs being targeted for tissue acquisition, depending on the endosonographer.

In addition to low agreement about LN characteristics, estimation of whether a specific LN is malignant exhibited only moderate accuracy. Overall accuracy for identifying malignant LNs was approximately 64%, with sensitivity ranging from 27% to 91% among individual endosonographers. Given that surgical resection remains the only curative option for patients with iCCA and pCCA 5 6 7 , accurately identifying malignant LNs is crucial for determining resectability. Although characteristics such as size (> 10 mm), round shape, sharp demarcation, and homogeneity are considered indicative of malignancy in mediastinal LNs, these criteria have not been conclusively validated for pancreato-biliary malignancies, including CCA 19 20 .

The present study further supports the notion that relying solely on LN morphology in EUS assessment is insufficient to reliably identify malignant LNs. Furthermore, the anatomical location of LNs is a significant factor in determining resectability. Presence of extraregional LNM generally warrants palliative treatment, whereas regional LNM may require a case-by-case evaluation regarding appropriateness of surgery 8 . However, distinguishing regional from extraregional LNs during EUS can be challenging, and this ambiguity complicates decisions about which LNs should be targeted for tissue acquisition. One objective feature that is less prone to interobserver variability is LN size. In light of this, LNs with a diameter > 10 mm should always be considered for biopsy 19 20 . Further research, such as that being conducted in the POELH trial, is necessary to clarify whether LNs measuring between 5 and 10 mm should also be targeted. The potential application of artificial intelligence (AI) in EUS-guided LN evaluation remains underexplored but could represent a future solution to current challenges in LN characterization 33 . Studies about AI for EUS-guided LN evaluation should take into account interobserver agreement by training the system on videos of LNs in which EUS-TA was performed, not based on assessment of individual endosonographers.

Several limitations should be acknowledged. First, pathological confirmation of LN status was available for only a subgroup of patients. Although the POELH trial protocol stipulated puncturing all LNs with a short-axis > 5 mm, fewer than half of these LNs were biopsied due to procedure constraints. This may have led to underestimation of the number of malignant LNs. In addition, use of static images for evaluating LN characteristics does not fully capture the dynamic nature of real-time EUS, potentially impacting accuracy of LN assessments. Follow-up studies on interobserver agreement on LN should aim to include videos and not still images. Finally, the low response rate (16.6%) and high proportion of expert participants may limit generalizability of the findings. It is likely that this selection bias affects the results and interpretability of this study. However, this may have resulted in an underestimation of interobserver variability rather than an overestimation, thereby reinforcing the robustness of the study conclusions, but not the findings themselves.

## Conclusions

In conclusion, this study underscores the significant challenges associated with visual interpretation of abdominal LN characteristics in patients with iCCA and pCCA. Even among expert endosonographers, interobserver variability was high, and accuracy in identifying malignant LNs based on morphology was moderate. These findings underscore the need for standardized criteria to guide LN tissue acquisition, and further research is needed to evaluate the role of AI in improving reliability of EUS-guided LN evaluation.

## Supplementary Material

Supplementary Material Zusatzmaterial

## References

[1] Blechacz B Komuta M Roskams T Clinical diagnosis and staging of cholangiocarcinoma Nat Rev Gastroenterol Hepatol 2011 8 512 522 10.1038/nrgastro.2011.131 21808282 PMC3331791

[2] DeOliveira ML Cunningham SC Cameron JL Cholangiocarcinoma: thirty-one-year experience with 564 patients at a single institution Ann Surg 2007 245 755 762 17457168 10.1097/01.sla.0000251366.62632.d3 PMC1877058

[3] Spolverato G Kim Y Alexandrescu S Is Hepatic resection for large or multifocal intrahepatic cholangiocarcinoma justified? Results from a multi-institutional collaboration Ann Surg Oncol 2015 22 2218 2225 10.1245/s10434-014-4223-3 25354576 PMC4834710

[4] Cillo U Fondevila C Donadon M Surgery for cholangiocarcinoma Liver Int 2019 39 143 155 10.1111/liv.14089 30843343 PMC6563077

[5] Radtke A Konigsrainer A Surgical therapy of cholangiocarcinoma Visc Med 2016 32 422 426 10.1159/000452921 28229077 PMC5290435

[6] Cai Y Cheng N Ye H The current management of cholangiocarcinoma: A comparison of current guidelines Biosci Trends 2016 10 92 102 10.5582/bst.2016.01048 27026485

[7] Weber SM Ribero D O'Reilly EM Intrahepatic cholangiocarcinoma: expert consensus statement HPB (Oxford) 2015 17 669 680 10.1111/hpb.12441 26172134 PMC4527852

[8] Blechacz B Cholangiocarcinoma: Current knowledge and new developments Gut Liver 2017 11 13 26 10.5009/gnl15568 27928095 PMC5221857

[9] Mavros MN Economopoulos KP Alexiou VG Treatment and prognosis for patients with intrahepatic cholangiocarcinoma: Systematic review and meta-analysis JAMA Surg 2014 149 565 574 24718873 10.1001/jamasurg.2013.5137

[10] Liang L Li C Jia HD Prognostic factors of resectable perihilar cholangiocarcinoma: a systematic review and meta-analysis of high-quality studies Ther Adv Gastrointest Endosc 2021 14 2631774521993065 10.1177/2631774521993065 PMC7882763 33629062

[11] Jolissaint JS Soares KC Seier KP Intrahepatic cholangiocarcinoma with lymph node metastasis: treatment-related outcomes and the role of tumor genomics in patient selection Clin Cancer Res 2021 27 4101 4108 33963001 10.1158/1078-0432.CCR-21-0412 PMC8282702

[12] Groot Koerkamp B Wiggers JK Allen PJ Recurrence rate and pattern of perihilar cholangiocarcinoma after curative intent resection J Am Coll Surg 2015 221 1041 1049 26454735 10.1016/j.jamcollsurg.2015.09.005 PMC4736142

[13] Ruys AT van Beem BE Engelbrecht MR Radiological staging in patients with hilar cholangiocarcinoma: a systematic review and meta-analysis Br J Radiol 2012 85 1255 1262 22919007 10.1259/bjr/88405305 PMC3487057

[14] Malikowski T Levy MJ Gleeson FC Endoscopic ultrasound/fine needle aspiration is effective for lymph node staging in patients with cholangiocarcinoma Hepatology 2020 72 940 948 31860935 10.1002/hep.31077

[15] Gleeson FC Rajan E Levy MJ EUS-guided FNA of regional lymph nodes in patients with unresectable hilar cholangiocarcinoma Gastrointest Endosc 2008 67 438 443 18061597 10.1016/j.gie.2007.07.018

[16] de Jong DM den Hoed CM Willemssen F Impact of endoscopic ultrasound in unresectable perihilar cholangiocarcinoma patients in liver transplantation work-up Gastrointest Endosc 2024 99 548 556 37890597 10.1016/j.gie.2023.10.047

[17] de Jong DM van de Vondervoort S Dwarkasing RS Endoscopic ultrasound in patients with resectable perihilar cholangiocarcinoma: impact on clinical decision-making Endosc Int Open 2023 11 E162 E168 36741342 10.1055/a-2005-3679 PMC9894690

[18] de Jong DM van de Vondervoort S Dwarkasing RS Endoscopic ultrasound with tissue acquisition of lymph nodes in patients with potentially resectable intrahepatic cholangiocarcinoma Endosc Int Open 2024 12 E998 E1005 39184062 10.1055/a-2366-2592 PMC11343620

[19] Catalano MF Sivak MV Rice T Endosonographic features predictive of lymph node metastasis Gastrointest Endosc 1994 40 442 446 10.1016/s0016-5107(94)70206-3 7926534

[20] Faige DO EUS in patients with benign and malignant lymphadenopathy Gastrointest Endosc 2001 53 593 598 10.1067/mge.2001.114060 11323584

[21] Takasaki Y Irisawa A Shibukawa G New endoscopic ultrasonography criteria for malignant lymphadenopathy based on inter-rater agreement PLoS One 2019 14 e0212427 10.1371/journal.pone.0212427 PMC6386303 30794598

[22] Yamamiya A Irisawa A Kashima K Interobserver reliability of endoscopic ultrasonography: Literature review Diagnostics (Basel) 2020 10 953 10.3390/diagnostics10110953 33203069 PMC7696989

[23] Quispel R van Driel LMJW Honkoop P Collaboration of community hospital endosonographers improves diagnostic yield of endoscopic ultrasonography guided tissue acquisition of solid pancreatic lesions Endosc Int Open 2019 07 E800 E807 10.1055/a-0898-3389 PMC6561772 31198843

[24] Sharma A Minh Duc NT Luu Lam Thang T A Consensus-Based Checklist for Reporting of Survey Studies (CROSS) J Gen Intern Med 2021 36 3179 3187 33886027 10.1007/s11606-021-06737-1 PMC8481359

[25] Castor EDC Castor electronic data capture. Amsterdam, The Netherlands: Ciwit BV https://castoredc.com

[26] Amin MB Greene FL Edge SB The Eighth Edition AJCC Cancer Staging Manual: Continuing to build a bridge from a population-based to a more "personalized" approach to cancer staging CA Cancer J Clin 2017 67 93 99 28094848 10.3322/caac.21388

[27] Johnson G Webster G Boškoski I Curriculum for ERCP and endoscopic ultrasound training in Europe: European Society of Gastrointestinal Endoscopy (ESGE) Position Statement Endoscopy 2021 53 1071 1087 10.1055/a-1537-8999 34311472

[28] Miyazaki M Ohtsuka M Miyakawa S Classification of biliary tract cancers established by the Japanese Society of Hepato-Biliary-Pancreatic Surgery: 3rd English edition J Hepatobiliary Pancreat Sci 2015 22 181 196 10.1002/jhbp.211 25691463

[29] Light RJ Measures of response agreement for qualitative data: Some generalizations and alternatives Psychol Bull 1971 76 365 377

[30] Hallgren KA Computing inter-rater reliability for observational data: An overview and tutorial Tutor Quant Methods Psychol 2012 8 23 34 10.20982/tqmp.08.1.p023 22833776 PMC3402032

[31] Landis JR Koch GG The measurement of observer agreement for categorical data Biometrics 1977 33 159 174 843571

[32] de Melo SW Panjala C Crespo S Interobserver agreement on the endosonographic features of lymph nodes in aerodigestive malignancies Dig Dis Sci 2011 56 3204 3208 10.1007/s10620-011-1725-8 21573731

[33] Khalaf K Terrin M Jovani M a comprehensive guide to artificial intelligence in endoscopic ultrasound J Clin Med 2023 12 3757 10.3390/jcm12113757 37297953 PMC10253269

